# Description of Copy Number Variations in a Series of Children and Adolescents with FASD in Reunion Island

**DOI:** 10.3390/children10040694

**Published:** 2023-04-07

**Authors:** Laëtitia Sennsfelder, Susie Guilly, Sébastien Leruste, Ludovic Hoareau, Willy Léocadie, Pauline Beuvain, Meïssa Nekaa, Maïté Bagard, Stéphanie Robin, Justine Lanneaux, Léa Etchebarren, Marilyn Tallot, Michel Spodenkiewicz, Jean-Luc Alessandri, Godelieve Morel, Maud Blanluet, Paul Gueguen, Bérénice Roy-Doray

**Affiliations:** 1 Laboratoire EPI (Etudes pharmaco-immunologiques), UFR Santé, Université de La Réunion, CHU (Centre Hospitalier Universitaire) de La Réunion, 97400 Saint-Denis, France; 2 Service de Génétique, CHU (Centre Hospitalier Universitaire) de La Réunion, La Réunion, 97400 Saint-Denis, France; 3 CIC 1410 (Centre d’Investigation Clinique), CHU (Centre Hospitalier Universitaire) de La Réunion, 97400 Saint-Denis, France; 4 UFR Santé, Université de La Réunion, 97410 Saint-Pierre, France; 5 Centre Ressources TSAF (Troubles du Spectre de l’Alcoolisation Fœtale), Fondation Père Favron, CHU (Centre Hospitalier Universitaire) de La Réunion, 97546 Saint-Pierre, France; 6 Centre Diagnostic TSAF (Troubles du Spectre de l’Alcoolisation Fœtale), CHU (Centre Hospitalier Universitaire) de La Réunion, 97400 Saint-Denis, France; 7 Pôle de Santé Mentale, CHU (Centre Hospitalier Universitaire) de La Réunion, 97448 Saint-Pierre, France; 8 Centre de Référence Anomalies du Développement et Syndromes Malformatifs Sud-Ouest Occitanie Réunion, Site Constitutif de La Réunion, 97400 Saint-Denis, France

**Keywords:** fetal alcohol spectrum disorder, copy number variation, chromosomal analysis on DNA chip

## Abstract

Background: Fetal Alcohol Spectrum Disorders (FASD) are the most common cause of neurocognitive impairment and social inadaptation, affecting 1 birth in 100. Despite the existence of precise diagnostic criteria, the diagnosis remains difficult, often confounded with other genetic syndromes or neurodevelopmental disorders. Since 2016, Reunion Island has been a pilot region for the identification, diagnosis, and care of FASD in France. Objective: To evaluate the prevalence and the types of Copy Number Variations (CNV) in FASD patients. Methods: A retrospective chart review of 101 patients diagnosed with FASD in the Reference Center for developmental anomalies and in the FASD Diagnostic Center of the University Hospital was performed. Records of all patients were reviewed to obtain their medical history, family history, clinical phenotype, and investigations, including genetic testing (CGH- or SNP-array). Results: A rate of 20.8% (n = 21) of CNVs was found including 57% (12/21) of pathogenic variants and 29% (6/21) of variants of uncertain signification (VUS). Conclusion: A particularly high number of CNVs was found in children and adolescents with FASD. It reinforces the plea for a multidisciplinary approach for developmental disorders to explore both environmental factors, such as avoidable teratogens and intrinsic vulnerabilities, especially genetic determinants.

## 1. Introduction

In 1968, Dr Paul Lemoine, a French pediatrician, described for the first time a large cohort of 127 children with neurodevelopmental disabilities, growth failure, and similar facial dysmorphism, all born to mothers who had consumed alcoholic beverages during their pregnancy [1]. Five years later, the same adverse effects of alcohol consumption during pregnancy were reported by Jones and Smith [2]. In 1996, the Institute of Medicine (IOM) established four distinct diagnostic categories: Fetal Alcohol Syndrome (FAS), partial Fetal Alcohol Syndrome (pFAS), Alcohol-Related Neurodevelopmental Disorders (ARND), and Alcohol-Related Birth Defects (ARBD) [3]. FAS constitutes the most complete and visible form and includes growth deficiency, characteristic distinctive facial features (such as short palpebral fissures, smooth philtrum, and thin upper lip), neurobehavioral impairment and structural brain alterations; pFAS associates with neurobehavioral impairment, characteristic facial features and, if prenatal alcohol exposure is unknown, growth deficiency or brain malformation. Characteristic facial features are absent and only neurobehavioral impairment is observed with a confirmed prenatal alcohol exposure in ARND. ARBD are mentioned when prenatal alcohol exposure is documented together with specific major malformations as described in animal models. Several guidelines such as the 4-digit code and Hoyme et al. classification updated in 2016 were proposed to help to diagnose FASD [4,5,6]. The risk and the severity of FASD can be explained by several factors such as the time, the dose, the frequency, and the duration of maternal alcohol consumption. These factors have an impact on the gravity of the prenatal alcohol effect, while the type of physical and neurobehavioral effects is influenced by the timing of exposure at different developmental stages, which differentiate FASD from other neurodevelopmental disorders. Also, maternal factors such as the age, the gravidity, the parity, and the nutritional status influence the severity of FASD [7].

Considered as the most frequent cause of avoidable neurocognitive disorders and social maladjustment, Fetal Alcohol Spectrum Disorders (FASD) remain a major public health concern [8] with a worldwide prevalence of 8 per 1000 children. The highest rates were found in South Africa (111 per 1000), Croatia (53 per 1000), Ireland (48 per 1000) and the United States (10 per 1000 children). Also, the global prevalence of FASD was estimated at 10.4 per 1000 persons in France [9]. A national estimate of the prevalence of FASD in newborns among all French regions was conducted by the French Public Health Agency in 2018 based on medico-administrative data (codes relating to fetal alcohol diagnosis) from the program for the medicalization of information systems (PMSI) in infants between 0 and 1 month of life: the overall FAS frequency was 0.48 per 1000 births in France between 2006 and 2013, but Reunion Island showed the highest prevalence with 1.22 per 1000 births—more than two and a half times the national rate [10]. On top of that, these prevalences are underestimated because of the complex diagnosis of children under 3 years since reliable neuropsychological testing cannot be performed. This underestimation is also due to the lack of knowledge of FASD by some practitioners, leading to a misdiagnosis. In Reunion Island, the awareness of health authorities and the mobilization of professionals have led to the implementation of a regional “FASD Action Plan” with the creation in 2016 of the FASD Resource Center for expertise, training, coordination, and centralization of information relating to the regional identification and monitoring of FASD [11]. One year later, the FASD Diagnostic Center was created to provide clinical services. The FASD Diagnostic Center offers a multidisciplinary diagnostic and functional assessment of children aged between 5 and 18 years and provides individualized recommendations for medical and socio-educational care. To date, more than 300 children have been diagnosed.

Unfortunately, the diagnosis of FASD remains complex, and this disorder is frequently underdiagnosed or misdiagnosed [12]. Its most visible form—FAS—shares clinical features with several genetic syndromes such as velocardiofacial syndrome (VCFS) also known as DiGeorge syndrome, Williams syndrome or Cornelia de Lange syndrome [13,14,15]. The same difficulties arise for the non-visible forms such as ARND, often confused with attention deficit hyperactivity disorder (ADHD) because both display executive function impairments, even if executive dysfunction is greater in FASD children compared to children with ADHD [16].

A genetic assessment is classically proposed as a first-line investigation in case of physical abnormalities, distinctive facial features, and/or neurodevelopmental disorders. Genomic alterations (Copy Number Variations: CNVs) have been previously reported in FASD [17,18,19].

The objective of this study was to evaluate the prevalence and the types of Copy Number Variations in children and adolescents diagnosed with FASD in Reunion Island.

## 2. Materials and Methods

A total of 101 children and adolescents highly suspected of FASD were evaluated between 2016 and 2022, using the 4-digit code. Birth parameters (weight, height, head circumference) were interpreted according to the audipog growth curves (https://www.audipog.net, accessed on 24 October 2022). Clinical and dysmorphological evaluation were performed either in consultation by a medical practitioner specialized in FASD and also medical director of the FASD Resource Center or as part of a multidisciplinary assessment at the FASD Diagnostic Center; genetic analyses including a chromosomal microarray analysis (CMA) by CGH-array or SNP-array and a molecular analysis for X-fragile syndrome were also systematically proposed. Before the age of 5, the diagnosis of FAS and pFAS was made on the notion of prenatal alcohol exposure (PAE) and the presence of specific facial dysmorphic features (thin upper lip, smooth philtrum, small palpebral features). The diagnosis of ARND was made only after 5 years when the assessment in the Diagnostic Center was performed with reliable psychological/neuropsychological testing. The 101 children and adolescents were all confirmed to have FASD.

DNA was isolated from peripheral blood samples using the chemagic 360 instrument (PerkinElmer^®^). The DNA concentration was normalized at 100 ng/μL. CGH-array was performed on SurePrint G3 Human CGH 60K microarray oligonucleotide (Agilent Technologies^®^, Santa Clara, CA, USA) with a resolution of 60 kb and cutoffs for the detection criteria of CNVs set at 400 kb. The CytoGenomics software (Agilent Technologies^®^, Santa Clara, CA, USA) was used for the analysis, utilizing the ADM-2 algorithm with a threshold of log2 ratio between −1 and 0.6. The SNP-array was performed on the Affymetrix CytoScan750K platform (Santa Clara, CA, USA). The cutoffs for the detection criteria for CNVs were set at 100 kb for genic regions and 400 kb for intergenic regions. The ChAS (Chromosome Analysis Suite^®^) software package (Santa Clara, CA, USA) was utilized for the analyses of SNP-array, using the Hidden Markov Model (HMM) with a threshold of log2 ratio between −0.45 and 0.3. Based on data from the genome version GRCh38 (hg38), the CEL files were analyzed with the Chromosome Analysis Suite v4.1 software. All CNVs were validated by FISH or QFM-PCR.

The results were interpreted and genotype–phenotype correlations were analyzed using public databases such as DGV (http://dgv.tcag.ca/dgv/app/home, accessed on 24 October 2022), OMIM (https://www.omim.org, accessed on 24 October 2022), DECIPHER (https://www.deciphergenomics.org, accessed on 24 October 2022), the International Standards of Cytogenetic Arrays (https://www.iscaconsortium.org/, accessed on 24 October 2022), the UCSC Genome Browser (http://genome.ucsc.edu/ last accessed on 24 October 2022), and PubMed (https://pubmed.ncbi.nlm.nih.gov, accessed on 24 October 2022). Copy number variations (CNVs) were interpreted and classified based on the consensus recommendation of the American College of Medical Genetics and Genomics (ACMG) [20], the Clinical Genome Resource (ClinGen), and the French Achropuce network guidelines (20220930-Aide-a-l-interpretation-CNV-revised-2022-VF.pdf) into five following categories:

− “Group 5”—pathogenic CNVs: these CNVs are associated with a consistent clinical phenotype described in multiple peer-reviewed publications with well-documented penetrance and expressivity; or CNVs overlapped completely with an established dosage-sensitive region; or multigenic CNVs in which at least one gene is known to be dosage sensitive. One subgroup was represented by genetic risk factors for neurodevelopmental disorders with Incomplete Penetrance and/or Variable Expressivity (“PIEV in French”, often inherited, which can be associated with variable and often not very specific phenotypes, making the prediction of the phenotype, and therefore genetic counseling, very difficult). These CNVs can shape the phenotype of the patient, but their pathogenicity could be influenced by a second event (double-”hit”) genetic, epigenetic, or environmental. Most of the time, this second “hit” is not identified, and even so, the modes of interaction between the two events and their phenotypic consequences are difficult to assess.
− “Group 4”—likely pathogenic including CNVs with strong evidence suggesting that they might ultimately be disease-causing, but without enough evidence available yet to definitively assert pathogenicity.
− “Group 3”—uncertain significance (VUS): corresponding to a broad category and including findings that are later described with additional evidence to be either pathogenic or benign.
− “Group 2”—likely benign corresponding to CNVs with strong evidence that they are likely not to be involved in Mendelian diseases, but not enough available evidence to state this definitively.
− “Group 1”—benign reported in multiple peer-reviewed publications or annotated in curated databases as benign variants. These CNVs should be documented in >1% of the population.

## 3. Results

### 3.1. Description of the Cohort

This series was divided into 41.6% (*n* = 42) patients with FAS, 34.6% (*n* = 35) patients with partial FAS and 23.8% (*n* = 24) patients with ARND. Children were mostly placed in the child welfare system (60.4%, *n* = 61), making it difficult to obtain information on maternal alcohol consumption. Prenatal alcohol exposure (PAE) was confirmed by the mother in 54.5% (*n* = 55) of cases, while it was highly suspected in 45.5% (*n* = 46) by the actors of the child welfare system or the other members of the family. Also, other toxics were associated with PAE, such as tobacco (32.7%, *n* = 33) and cannabis (5.9%, *n* = 6).

### 3.2. Genetic Analyses

The fragile-X analysis was performed in 69 patients and normal for all (100%). Concerning genomic analysis, a CNV was observed in 20.8% (*n* = 21) of cases divided in 7 pathogenic CNVs of group 5 (including 5 neurodevelopmental susceptibility factors with incomplete penetrance and variable expressivity), 5 likely pathogenic CNVs (group 4), 6 VUS (group 3), 1 likely benign CNV (group 2) and 2 benign CNVs (group 1) (Table 1). The inheritance of all pathogenic and likely pathogenic CNVs was not known since parental samples were not available. It was also the case for most of VUS except for 17q25.3 deletion and 5q22.1 duplication.

**Table 1:** Description of clinical phenotype and CNVs found in our series of FASD patients.

Case Number	Sex	Prenatal Alcohol Exposure (PAE)	Age at Definitive Diagnosis (Years)	Weight (Percentile)	Height (Percentile)	Head Circumference (Percentile)	Clinical Features	Cognitive Deficit	Verbal Comprehension	Fluid Reasoning	Working Memory	Processing Speed	CNV	Size (Mb)	Type	Classification	Genes of Interest (Brain Structure or Brain Functions)	Diagnosis
1487	M	Chronic	7	>10th	>10th	>3rd	Short palpebral fissures, nasal root hypoplasia, short philtrum	73	81	80	80	82	1q21.1q21.2 (146506310_147824207)	1.15	deletion	IPVE	*HYDIN2*, *GJA5*	ARND
10162	M	NA ^1^	16	<10th	<10th	<3rd	Short palpebral fissures, long flat philtrum, thin upper lip	NA ^2^	NA ^2^	NA ^2^	NA ^2^	NA ^2^	1q21.1q21.2 (146476526_148000829)	1.5	deletion	IPVE	*GJA5*, *GJA8*	FAS
319	F	Chronic	17	<10th	<10th	<3rd	Hypertelorism, thin upper lip, smooth philtrum, brachydactyly	65	67	76	66	75	16p11.2(29673954_30198600)×1	0.525	deletion	IPVE	*QPRT*, *DOC2A*, *TAOK2*, *ALDOA*, *PRRT2*	FAS
324_1	M	Acute	7	>10th	10th	>3rd	Retrognathism, unilateral cryptorchydia	83	89	85	91	123	16p12.2(217398862,21951379_22380197x1,22645705×2)	0.429	deletion	IPVE	*CDR2*	ARND
324_2	M	Acute	5	>10th	>10th	>3rd	Smooth philtrum, thin upper lip, short nails	65	69	77	78	69	16p12.2(21739886×2,21951379_22380197×1,22645705×2)	0.429	deletion	IPVE	*CDR2*	pFAS
1006	M	Chronic	6	>10th	>10th	>3rd	Short palpebral fissures, thin upper lip, smooth philtrum, camptodactyly, short nails	60	54	65	72	74	2q31.1(172608208×2,172666705_173038935×1,173197025×2)	0.380	deletion	Pathogenic	*RAPGEF4*	pFAS
1004	F	Chronic	3	<10th	<10th	<3rd	Thin upper lip, retrognathism, low-set ears	NA ^2^	NA ^2^	NA ^2^	NA ^2^	NA ^2^	Xq21.1q28(82,335,645×2,82,461,036-155,232,907×1)	72	deletion	Pathogenic		FAS
7241_1	M	NA ^1^	9	<10th	<10th	<3rd	Short palpebral fissures, thin upper lip	52	65	67	65	56	2p16.2(52942477_54649776)×1	1.7	deletion	Likely pathogenic	*SPTBN1*	FAS
7241_2	M	NA ^1^	5	<10th	<10th	<3rd	Smooth philtrum, thin upper lip, camptodactyly, hypertelorism	NA ^2^	89	95	100	94	2p16.2(52942477_54649776)×1	1.7	deletion	Likely pathogenic	*SPTBN1*	FAS
7241_3	F	Acute	8	<10th	<10th	>3rd	camptodactyly	NA ^2^	NA ^2^	NA ^2^	NA ^2^	NA ^2^	2p16.2(52917002_54649776)×1	1.7	deletion	Likely pathogenic	*SPTBN1*	ARND
7241_4	F	Acute	9	NA	NA	NA	Smooth philtrum, camptodactyly	NA ^2^	70	106	85	95	2p16.2(52917002_54649776)×1	1.7	deletion	Likely pathogenic	*SPTBN1*	pFAS
7241_5	F	NA ^1^	6	>10th	>10th	>3rd	Hypertelorism, nasal root hypoplasia, nostril anteversion, flat philtrum, thin upper lip, camptodactyly	NA ^2^	72	83	90	97	2p16.2(52917002_54649776)×1	1.7	deletion	Likely pathogenic	*SPTBN1*	pFAS
178	M	Acute	12	<10th	NA	NA	camptodactyly	76	73	88	76	92	17q25.3(80529730×2,80561130_81029×1)	0.469	deletion	VUS	*FOXK2*	ARND
6256	M	Chronic	3	<10th	<10th	<3rd	Smooth philtrum, thin upper lip, camptodactyly, clinodactyly	NA ^2^	NA ^2^	NA ^2^	NA ^2^	NA ^2^	1q32.1(204097859_204946764)×3	0.849	duplication	VUS	*PPP1R15B*, *NFASC*	FAS
326	M	NA ^1^	18	<10th	<10th	<3rd	No	57	67	70	63	61	7q21.11(204097859_204946764)×3	0.263	duplication	VUS	*PCLO*	ARND
9219	M	Chronic	16	<10th	<10th	>3rd	Short palpebral fissures, thin upper lip, smooth philtrum	NA ^2^	NA ^2^	NA ^2^	NA ^2^	NA ^2^	8q23.3(112972853_113128118)×1	0.16	deletion	VUS	*CSMD3*	FAS
3918	M	NA ^1^	4	<10th	<10th	<3rd	Short palpebral fissures, smooth phitrum, nasal wings hyperplasia	NA ^2^	NA ^2^	NA ^2^	NA ^2^	NA ^2^	5q22.1(109,813,483×2,109,851,548-111,194,538×3,111,370,920×2)	1.35	duplication	VUS	*NREP*	FAS
5930	F	Binge	5	>10th	>10th	>3rd	Short palpebral fissures, smooth philtrum, thin upper lip	NA ^2^	NA ^2^	NA ^2^	NA ^2^	NA ^2^	4q12(53416699_53631029)×3	0.214	duplication	VUS	*LNX1*	pFAS
2333	M	Acute	7	>10th	<10th	>3rd	Thin upper lip	NA ^2^	76	88	76	98	Xq28(149386745×1,149410730_149709802x0,149750397×1)	0.299	deletion	Likely benign		ARND
429	F	Acute	6	>10th	<10th	>3rd	Smooth philtrum, thin upper lip	NA ^2^	62	58	NA ^2^	NA ^2^	19p13.11 (17927322_18052605)×3	0,125	duplication	benign		FAS
334	F	Acute	5	<10th	<10th	<3rd	Smooth philtrum, thin upper lip, nasal root hypoplasia	62	57	74	82	88	5q14.3(88450293_90077119)×3	1.63	duplication	benign		FAS

^1^ Data not available due to FASD children in the child welfare system. ^2^ Data not available or absence of assessment at the FASD Diagnostic Center.

#### 3.2.1. Pathogenic CNVs (Neurodevelopmental Susceptibility Factors)

The recurrent **1q21.1-q21.2 deletion** was found in two patients (case numbers 1487 and 10162) diagnosed with ARND and FAS, respectively. Among this deletion, 3 genes of interest (*HYDIN2*, *GJA5*, *GJA8*) were reported. In our series of patients, patient #1487 was classified as ARND because of the absence of growth retardation and clinical facial features of FAS, but cognitive level at the lower range with typical neuropsychological profile and established chronic prenatal exposure to alcohol. On the contrary, patient #10162 classified as FAS presented with growth retardation, psychomotor delay and typical FAS dysmorphism. Both had neither eye abnormalities, especially cataract, nor heart abnormalities classically described in the 1q21.1-q21.2 deletion. Cognitive difficulties including impaired working memory or processing speed were observed for patient #1487 with ARND. The functional assessment in the FASD Diagnostic Center has not yet been realized for patient #10162 with FAS.

A **16p11.2 deletion** was reported in a patient with FAS (case number 319), including 5 genes of interest (*QPRT*, *DOC2A*, *TAOK2*, *ALDOA*, *PRRT2*). The patient was classified as FAS because of the association of growth retardation, a characteristic dysmorphism, a cognitive deficiency with autistic traits in a context of a chronic prenatal exposure to alcohol.

A **16p12.2 deletion** was observed in two siblings (case numbers 324_1 and 324_2) with an acute prenatal alcohol exposure classified as ARND and partial FAS, respectively. The *CDR2* gene was included in this deletion. Neither patient had growth retardation. Patient #324_2 presented with a typical FAS dysmorphism. Patient 324_2 had cognitive deficiency with difficulties in verbal comprehension, fluid reasoning, working memory, and processing speed. Patient #324_1 had no cognitive deficiency, but school difficulties, and attention disorders.

#### 3.2.2. Other Pathogenic CNVs

A **2q31.1 deletion** was reported in one patient (case number 1006) with partial FAS. In this deletion, *RAPGEF4* was reported as a gene of interest. Our patient exhibited a mild mental deficiency and a typical FAS dysmorphism with short palpebral fissures, smooth philtrum, thin upper lip, camptodactyly of fingers and short nails. He had neither growth retardation nor microcephaly.

A **Xq21.1-q28 deletion** was reported in a girl with FAS (case number 1004). The girl presented with postnatal motor and language delay, growth retardation, ventricular septal defect, hypertelorism, flat philtrum, thin upper lip, low-set ears, and increased nipple spacing.

The maternal alcohol consumption for these cases was chronic during the pregnancy.

#### 3.2.3. Likely Pathogenic CNVs

The likely pathogenic CNV **2p16.2 deletion** including the *SPTBN1* gene was described in 5 younger siblings classified as FAS, partial FAS, and ARND. Patients with FAS (case numbers 7241_1, 7241_2, 7241_5) presented with growth retardation and typical distinctive facial traits. Patient #7241_1 had a mild cognitive deficit. Patients #7241_2 and #7241_5 had the same difficulties. The information of cognitive deficit was not yet available for the rest of the siblings. Patient #7241_3 was diagnosed with ARND and had growth retardation, a camptodactyly, but neither facial dysmorphism nor microcephaly. She had school difficulties with poor working memory and attention deficit. Patient #7241_4 was diagnosed as partial FAS: he had a smooth philtrum, and camptodactyly; he had school difficulties with selective alteration in verbal comprehension. For these 5 siblings, the mother declared an acute alcohol consumption during pregnancy only for patients #7241_3 and #7241_4. This prenatal alcohol consumption was associated with a chronic paternal preconceptional consumption.

#### 3.2.4. Variant of Uncertain Significance (VUS)

An inherited **17q25.3 deletion** was reported in a patient with ARND (case number 178). *FOXK2* was reported as a gene of interest. This deletion was inherited from the mother. Our patient had developmental and language delays. He had no cardiac defect. A cognitive deficit was not observed, but the patient had mild difficulties, especially in verbal comprehension and working memory. The mother did not have a cognitive assessment but had a history of moderate school difficulties and an acute alcohol consumption during the pregnancy.

A **1q32.1 duplication** including two genes of interest (*PPP1R15B*, *NFASC*) was described in a patient with partial FAS (case number 6256) with a chronic PAE. The literature reports clinical cases with a 1q32.1 duplication but of different sizes [15,16]. Our patient diagnosed with FAS had learning deficit, growth retardation, and typical dysmorphism such as smooth philtrum, thin upper lip, camptodactyly and clinodactyly.

A **7q21.11 duplication** comprising the *PCLO* gene was reported in an ARND patient (case number 326) with mild mental deficiency and growth retardation but without dysmorphism.

A **8q23.3 deletion** partially comprising the *CSDM3* gene was reported in a patient with FAS (case number 9219). This patient had short palpebral fissures, a smooth philtrum, thin upper lip, growth retardation, but no microcephaly. The prenatal alcohol consumption was classified as chronic.

A paternal inherited **5q22.1 duplication** was observed in a FAS patient diagnosed under 5 years (case number 3918). This duplication, including 6 genes such as the *NREP* gene implicated in neuronal functions, was considered as pathogenic and associated with intellectual disability and neuropsychiatric disorders. This patient had a growth retardation and a typical FAS dysmorphy.

A **4q12 duplication** classified as VUS was reported in a partial FAS patient (case number 5930). He had learning deficit with altered working memory, a typical FAS dysmorphism, but no growth retardation or microcephaly. A binge alcohol consumption was reported by the mother during the pregnancy.

The cognitive data were not available for all these patients with a variant of uncertain significance due to the absence of functional assessment at the FASD Diagnostic Center.

## 4. Discussion

This article is the second publication from a relatively large cohort of confirmed patients with FASD together with genetic analyses [17]. To our knowledge, it is the only study performed in a French FASD population. It differs from the previous studies carried out on cohorts of patients referred to a Department of Genetics with the knowledge of prenatal alcohol exposure and a suspected diagnosis of FASD, sometimes not confirmed [18,19].

The results of this study suggest that patients with FASD can also be carriers of rare CNVs, often impacting important neurodevelopmental genes and thus accumulating vulnerabilities.

A rate of 20.8% (21/101) of CNVs was found in this series. Among these 21 CNVs, 12 were classified as pathogenic or likely pathogenic, and contributing to the phenotype; 6 were classified as VUS with possible implication, especially since the genes of interest included in the rearrangement are associated with developmental or neurological functioning. While most of these CNVs have been described in the literature, this is the first description of these CNVs in a FASD population. This rate was higher than in the previous study of Jamuar et al. published in 2018. They described a study pointing at the interest of genetic analyses in patients with a suspicion of FASD. This study was conducted on 36 children addressed at the Boston Children’s Hospital Genetics Clinic with a suspicion of FASD. A pathogenic CNV was found in 14.3% of cases of FASD patients [19]. In 2017, the study performed by Zarrei et al. among 95 Canadian children with FASD demonstrated a rate of CNVs of 15% (*n* = 14 out of 95), but methodological differences make it impossible to compare the two rates [17]. However, these CNVs were described by the authors: (1) as impacting genes implicated in brain functions and described in autism spectrum disorders and other neurodevelopmental disorders, or (2) were located in regions associated with known genomic disorders. Among these fourteen CNVs, twelve might possibly contribute to the FASD phenotype [17]. One of the limitations of this study is the absence of a control group to determine the prevalence of CNVs in the Reunionese population. However, an estimated rate of a genome-wide de novo CNV was established as 1.2 × 10^−2^ events per transmission per generation (95% CI 5.3 × 10^−3^–2.2 × 10^−2^), which is consistent with the estimation of other studies (0.5–3%) [21,22].

In this study, a pathogenic CNV was found in the subchromosomal region 1q21.1, which is considered as one of the hotspots in the human genome for deletions and reciprocal duplications [23,24,25]. The 1q21.1 deletion is implicated in numerous developmental disorders including schizophrenia, intellectual disability, developmental delay, speech problems, autism spectrum disorders, motor impairment, and epilepsy. It also included craniofacial abnormalities, cataracts, and cardiac anomalies. The clinical phenotype included growth delay and microcephaly. Anomalies in fine motor skills, working memory, complex processing speed, and visual attention were also reported.

Another pathogenic CNV was found in the region of 2q31.1. In contrast to the subchromosomal region of 1q21.1, microdeletions in the region of 2q31.1 are rare. According to the literature, the 2q31.1 deletion is a well-known syndrome characterized by developmental delay, growth retardation, seizures, craniofacial dysmorphism including microcephaly, short palpebral fissure, broad eyebrows with lateral flare, low-set ears with thickened helices and lobules, and micrognathia. Congenital anomalies are also reported such as limb abnormalities including ectrodactyly/monodactyly, syndactyly, brachydactyly, camptodactyly, clinodactyly; heart defects; genital anomalies; craniosynostosis [24,25,26,27,28].

The last pathogenic CNV found was a Xq21.1-q28 deletion, which constitutes an equivalent of Turner Syndrome [29].

The likely pathogenic CNV described in this study includes the *SPTBN1* gene. This gene is implicated in growth delay, cognitive deficits and behavioral abnormalities such as attention/concentration disorder [30,31].

Concerning the VUS of this study, the 17q25.3 deletion, either inherited or de novo are usually associated with heart disease, developmental, and language delays [32]. Also, the *CSDM3* gene included in the 8q23.3 deletion is a candidate gene for autism spectrum disorders and myoclonic epilepsy [33,34].

In this series of patients with abnormal (pathogenic, likely pathogenic, and VUS) CNVs, almost half of them (8/18, 44.4%) were reported in patients with a complete form of FAS. This suggests that the associated genetic abnormality may be responsible for a more severe phenotype. Most of the abnormal CNVs reported in this study could induce developmental disorders, growth retardation, congenital malformations, and consequently reinforces embryofetal developmental vulnerability. For example, the girl with the deletion Xq21-q28 exhibited a more severe growth deficiency and cardiac malformations not present in her twin sister with ARND but not a carrier of the Xq deletion. Similarly, the girl with the 16p11.2 microdeletion (CNV of susceptibility to neurodevelopmental disorders, with incomplete penetrance and variable expressivity) presented with more severe autistic symptoms than commonly seen in FASD and, in this case, more severe than his younger brother.

Nevertheless, this associated genetic abnormality might also change the phenotype, particularly morphological, which can make it more difficult to assess the typical dysmorphy of FAS and lead to a bad classification from FAS to ARND.

The association between prenatal alcohol exposure and abnormal CNVs in patients with FASD has not been studied yet. The coexistence may be a coincidence and it is not surprising to find CNVs in a population with neurodevelopmental disorders. Nevertheless, we hypothesize a possible link between alcohol consumption and the occurrence of chromosomal microrearrangements. The harmful effects of alcohol on epigenetic mechanisms are increasingly being studied [35,36,37,38,39,40,41], which concerns abnormal DNA methylation, modifications of non-coding RNA, but also chromatin abnormalities. Could post-transcriptional modifications of histones [41] consecutive to alcohol exposure lead to a fragility of chromosomal regions and to chromosomal breaking?

In conclusion, this study is the first French study carried out from a large cohort of patients with FASD. With a proportion of genomic anomalies higher than those reported in the international literature, it confirms the importance of systematically completing the investigations carried out during the assessment of a FASD by a genetic assessment, both clinical and biological, with CMA. This genetic assessment is fundamental in order to better understand the interactions between genetic inheritance and neurotoxic teratogens, and to propose an appropriate malformative assessment and follow-up, as well as optimal tertiary prevention combining information on alcohol consumption and genetic counseling.

More generally, this study reinforces the plea for a global and multidisciplinary approach to embryonic developmental disorders, with etiological investigations for both environmental factors, such as avoidable teratogens like alcohol or drugs, and intrinsic vulnerabilities, in particular those genetically determined.

## Data Availability

Individual data are unavailable due to privacy restrictions.
